# Switchable wetting of oxygen-evolving oxide catalysts

**DOI:** 10.1038/s41929-021-00723-w

**Published:** 2021-12-30

**Authors:** Tzu-Hsien Shen, Liam Spillane, Jiayu Peng, Yang Shao-Horn, Vasiliki Tileli

**Affiliations:** 1 Institute of Materials, École Polytechnique Fédérale de Lausanne, Lausanne, Switzerland; 2 Gatan Inc., Pleasanton, CA USA; 3 Department of Materials Science and Engineering, Massachusetts Institute of Technology, Cambridge, MA USA; 4 Department of Mechanical Engineering, Massachusetts Institute of Technology, Cambridge, MA USA; 5 Research Laboratory of Electronics, Massachusetts Institute of Technology, Cambridge, MA USA

**Keywords:** Electrocatalysis, Energy

## Abstract

The surface wettability of catalysts is typically controlled via surface treatments that promote catalytic performance. Here we report on potential-regulated hydrophobicity/hydrophilicity at cobalt-based oxide interfaces with an alkaline solution. The switchable wetting of single particles, directly related to their activity and stability towards the oxygen evolution reaction, was revealed by electrochemical liquid-phase transmission electron microscopy. Analysis of the movement of the liquid in real time revealed distinctive wettability behaviour associated with specific potential ranges. At low potentials, an overall reduction of the hydrophobicity of the oxides was probed. Upon reversible reconstruction towards the surface oxyhydroxide phase, electrowetting was found to cause a change in the interfacial capacitance. At high potentials, the evolution of molecular oxygen, confirmed by operando electron energy-loss spectroscopy, was accompanied by a globally thinner liquid layer. This work directly links the physical wetting with the chemical oxygen evolution reaction of single particles, providing fundamental insights into solid–liquid interfacial interactions of oxygen-evolving oxides.

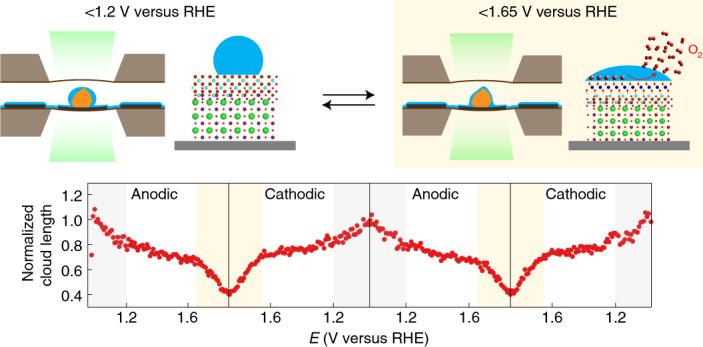

## Abstract (web summary)

Surface treatments can tune catalysts’ wettability, which can be used to promote their catalytic performance. Now, a potential-dependent dynamic wetting behaviour of cobalt-based oxide catalysts is identified before and during the oxygen evolution reaction.

## Main

Switchable wetting properties of solid surfaces from a hydrophobic to a hydrophilic character are of great interest for stimuli-responsive smart materials. In particular, the solid–liquid interface between a functional inorganic material and a liquid is exploited in a wide range of technological applications spanning from electrochemical energy systems^1^, water purification^2^, tunable optical lenses^3^, self-cleaning surfaces^4,5^ and sensors^6^. With respect to metal oxides, a reversible wettability can result in structural or chemical modifications induced by a variety of stimuli including light illumination^7,8^, electrical potential^9–12^ and temperature^13^. Potential-induced wettability of metal oxides proceeds through electrowetting^11,14^ especially for dielectric oxides such as Al_2_O_3_ (ref. ^15^) and TaO_*x*_ (ref. ^16^). When the potential is applied, the charges are accumulated at the interface and are arranged in the form of a capacitor, resulting in reduction of the surface tension at the solid–liquid interface^11^. Additionally, the surface chemistry of metal oxides can electrochemically change, and therefore the wettability of metal oxides can be altered via redox reactions. For example, it has been reported that the hydrophobic Cu_2_O surface reduces to hydrophilic metallic copper when a cathodic potential is applied^10^.

In practice, the wettability of solid surfaces under an applied potential can play a critical role in energy conversion^17–19^ and especially with respect to heterogeneous catalysis^20,21^. Hydrophobic surfaces have also been reported to promote CO_2_ reduction on a copper surface^22^. Moreover, studies showed that enhancement of the hydrophilicity of NiFe hydroxide by phosphorylation^17^ or plasma treatments^18^ can increase the activity of the oxygen evolution reaction (OER)—the anodic, sluggish reaction in water splitting^23^. Hydrophilicity of the surfaces has been shown to promote the charge-transfer rate between the electrolyte and electrode, enhancing OER activity^18^, whereas perovskite oxides with more hydrophobic surfaces have been shown to be more active for the oxygen reduction reaction (ORR)^19^. It becomes evident that a fundamental understanding of the potential-regulated wetting of catalytic particles could unlock its effect on their activity and stability.

To characterize the wetting character of catalysts, analyses in planar structures are mainly performed^17^. The most common technique involves measurement of the contact angle between a liquid droplet and a solid surface, which is related to interfacial tension^24^. During such measurements, optical images of droplets on thin-film surfaces are taken at a fairly bulk scale (approximately several hundred microns)^25^. Specifically for determining the electrowettability, electrical impedance spectroscopy (EIS) is used to acquire information about the relationship between accumulated charges and wetting under an electric potential^26^. More surface-sensitive techniques such as X-ray photoelectron spectroscopy have been used to study the relationship between wetting and surface state especially in the case of water-splitting catalysts^19^. Liquid-phase transmission electron microscopy (TEM) shows promise for exploring these effects on a single-particle level^27^.

In this study we performed operando TEM in liquids to probe the dynamic wetting behaviour of oxygen-evolving cobalt-based oxide catalysts under potential cycling, including probing of the highly OER-active perovskite Ba_0.5_Sr_0.5_Co_0.8_Fe_0.2_O_3−*δ*_ (BSCF), where *δ* denotes the number of oxygen vacancies. The potential-dependent variation of the contrast, which indicates the movement of the surrounding alkaline solution in the images, is associated with the modification of the wettability at the oxide surfaces. Overall, when an anodic potential is applied, the hydrophobicity of the oxides reduces due to electrowetting and OH^−^ accumulation at the surfaces. The reduction of hydrophobicity is stabilized after formation of the hydrophilic oxyhydroxide phase on BSCF and spinel Co_3_O_4_ at a potential of ∼1.2 V versus the reversible hydrogen electrode (RHE), which is related to the Co^2+^/Co^3+^ redox reaction. At anodic applied potentials higher than 1.6 V versus RHE, electron energy-loss spectroscopy (EELS) confirms the evolution of O_2_ that leads to a thinner liquid environment.

## Results

### Operando TEM observation of BSCF under potential cycling

**Fig. 1:**
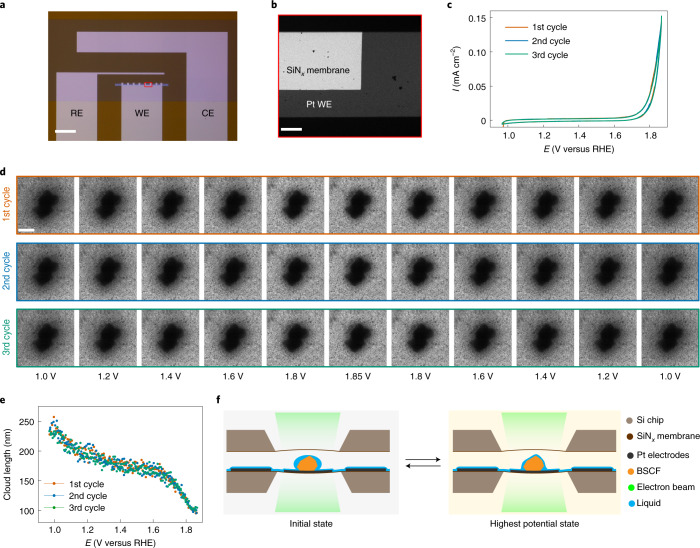
Real-time monitoring of a BSCF particle during CV measurements. **a**, Optical microscopy image of a customized electrochemical chip with three platinum thin-film electrodes: RE, reference electrode; WE, working electrode; CE, counter electrode. Scale bar, 250 μm. **b**, Close-up BF-TEM image of the WE loaded with BSCF particles. Scale bar, 5 μm. **c**, Polarization curves of three CV cycles in 0.1 M KOH in a liquid-cell enclosure. Scan rate, 20 mV s^−1^. **d**, BF-TEM images at different potential stages for the first, second and third cycles. Acquisition was done at a dose rate of 3 *e*^−^ Å^−^^2^ s^−1^. Scale bar, 400 nm. **e**, Cloud length in each frame of the TEM images as a function of applied potential (versus RHE). **f**, Schematic of reversible liquid movement surrounding BSCF particles in a liquid-cell enclosure. Shown are the initial state (0 V versus RHE) when the hydrophobic surface character of the BSCF surface accumulates liquid, and the final state (1.85 V versus RHE) where a more hydrophilic surface character is probed. All potential values are reported versus RHE. Source data

BSCF particles were dropcast on customized electrochemical chips patterned with three platinum thin-film electrodes as shown in Fig. 1a. The operando TEM measurements were performed on a BSCF particle (dark contrast) that sits on the platinum working electrode, ensuring the electrochemical connection of the system (Fig. 1b). Bright-field (BF) TEM images were acquired during three cycles of cyclic voltammetry (CV) ranging from 1.0 to 1.85 V versus RHE (Fig. 1c) using an alkaline solution of 0.1 M KOH as the electrolyte. Figure 1d shows representative BF-TEM images of the particle’s response to the three CV cycles (the full sequence can be seen in Supplementary Movie 1). Interestingly, a reversible phenomenon of a dense cloud surrounding the particles and subsequent dissolution of the cloud was observed which is coupled with the potential cycling. In particular, the cloud formation substantially diminished during the anodic scan and increased during the reverse cathodic scan. Image processing consisting of thresholding and segmentation was applied to determine the modification of the contrast from the BF-TEM images (see Methods for details). An example of the postprocessed segmentation of the first frame is shown in Supplementary Fig. 1. A quantitative calculation of the cloud length that surrounds the particle plotted as a function of the applied potential is depicted in Fig. 1e. The figure illustrates that the values of cloud length decrease and reversibly increase under potential cycling. This long-range alteration of the environment surrounding the BSCF particles is around several hundred nanometers, and hence the cloud cannot be associated with the direct imaging of the electrical double layer which is at most several nanometers^28^. Additionally, this phenomenon probed in liquid-phase TEM is inherently different from the structural oscillations of BSCF particles induced by the electron beam in water vapour, as previously reported^29^. In our case, the operando measurements were performed at a fairly low electron beam dose (3 *e*^−^ Å^−^^2^ s^−1^) to deter electron beam irradiation-induced effects.

We believe that this long-range alteration is associated with the fluctuation of the liquid that wets the BSCF particles in the presence of the liquid electrolyte. The ability to produce such contrast from the liquid solution implies that the TEM liquid cell is not completed filled with the electrolyte and the particles are not fully immersed in liquid. In practice, the electrolyte forms a layer across the electrodes and the particles, as shown in the schematics of Fig. 1f. The contrast in BF-TEM is due to the amplitude difference between the transmitted and scattered electrons. The difference in liquid thickness generates the contrast in BF-TEM which thus indicates the dynamic movement of the liquid during cycling. It should be noted that bulging of the membranes would not lead to such locally induced amplitude contrast. When the potential sweeps to lower values, the liquid surrounding the particles becomes thicker and generates the contrast of the cloud, whereas at higher anodic potentials, the contrast around the surface of the BSCF particles disappears due to the thinner liquid that wets the surface of the particles. This pronounced potential-dependent liquid movement is attributed to the surface functionalization of the BSCF particles. That is, the wettability of the BSCF surface triggers the liquid movement and hence the amplitude contrast of the cloud surrounding the particles changes in the BF-TEM images. For all three cycles, at the initial state, the surfaces display a hydrophobic character (Fig. 1f, left), whereas at the highest potential state, the surface of the particles exhibits a hydrophilic character (Fig. 1f, right). Remarkably, Fig. 1e illustrates that BSCF exhibits identical reversible wetting for each cycle stimulated by the application of the potential. Furthermore, at the anodic potential of ∼1.65 V versus RHE an abrupt release of the cloud surrounding the particles was observed. This depletion of the liquid at this potential range appears to be related to the OER take-off potential, as seen in Fig. 1c.

### Operando EELS analysis of molecular oxygen evolution

**Fig. 2:**
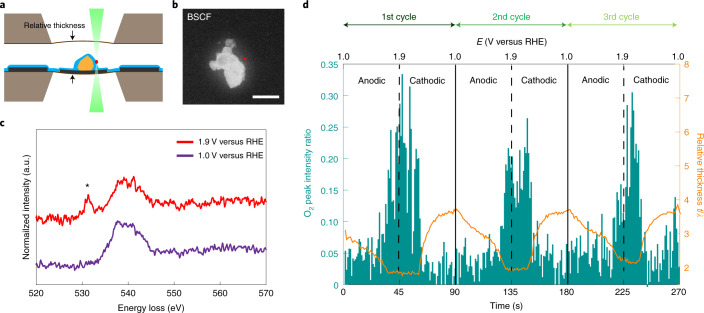
Operando EELS analysis of molecular oxygen evolution in close proximity to BSCF particles. **a**, Schematic of STEM-EELS probing near BSCF particles in a liquid-cell enclosure. **b**, High-angle annular dark-field STEM image of BSCF particles in a liquid-cell enclosure. The red point indicates the position of the electron beam during EELS acquisition. Scale bar, 1 μm. **c**, Oxygen K EEL spectrum. The asterisk at 531 eV indicates the peak feature that results from molecular oxygen. **d**, Plot of O_2_ peak intensity ratio (green) and relative thickness (orange curve) as a function of elapsed time (bottom axis) and applied potential (top axis) corresponding to CV measurements in Supplementary Fig. 2. The relative thickness is defined on the basis of low-loss EEL spectra (Supplementary Fig. 4). Source data

To corroborate that the evolution of oxygen can lead to this abrupt dissipation of the cloud surrounding the particles at ∼1.65 V versus RHE, operando EELS in scanning TEM (STEM) mode was performed in the liquid cell during potential cycling. The evolution of molecular oxygen during the OER can be probed by monitoring the oxygen’s core-loss K edge. Overall, the oxygen K edge in EELS is associated with the transition of the oxygen 1*s* to unoccupied 2*p* states. For the binary O_2_ molecule, the signature peak feature in the oxygen K EEL spectra is at an energy-loss of 531 eV and it depicts the transition of oxygen 1*s* → π* (ref. ^30^). To record the evolving molecular oxygen in relation to the applied potential in real time, we probed the environment in close proximity to the particle–liquid interface as shown in the schematic illustration in Fig. 2a. The convergent electron-beam was placed next to the BSCF particle (shown by the red dot in the annular dark-field image in Fig. 2b) to avoid electron-beam-induced irradiation effects. EEL spectra were continuously acquired during CV (Supplementary Fig. 2) to monitor the evolution of the O_2_ peak. Figure 2c shows the acquired oxygen K EEL spectra at 1.0 V and 1.9 V versus RHE from the first cycle. The distinct peak feature at 531 eV in the spectrum at 1.9 V indicates molecular oxygen evolution, whereas the broad peak at 540 eV appearing in all oxygen K EEL spectra is related to the background signal that comes from the KOH solution and the SiN_*x*_ membranes. To evaluate the appearance of molecular oxygen, we divide the O_2_ peak area at 531 eV with respect to the broad peak area at 540 eV and define this as O_2_ peak intensity ratio (more information on the calculation can be found in Supplementary Fig. 3). The O_2_ peak intensity ratio evolves periodically as a function of elapsed time, as shown in Fig. 2d, which can be correlated with the potential during the CV measurements. This observation unambiguously confirms that the change of the cloud length at ∼1.65 V versus RHE in Fig. 1e is related to O_2_ evolution.

At the same time, the specimen thickness (*t*) in units of the local inelastic mean free path (*λ*) varies inversely with the relative O_2_ peak intensity (overlaid in Fig. 2d). The relative thickness includes information that has to do with the SiN_*x*_ membranes, liquid and electrodes in the direction parallel to the electron beam (as sketched in Fig. 2a). The relative thickness (*t*/*λ*) is determined by the intensity ratio of the zero-loss peak to the total EEL spectrum as shown in Supplementary Fig. 4. The fluctuation of the relative thickness confirms the dissolution of the cloud and indicates that the liquid becomes thinner when oxygen is being evolved. Additionally, similar operando EEL measurements were performed on Co_3_O_4_ as shown in Supplementary Fig. 5. In this case, the relative O_2_ intensity also varies periodically with molecular oxygen evolving at high anodic potential. Finally, it is noted that this periodic evolution of relative O_2_ intensity or relative thickness did not occur on a pure platinum working electrode (Supplementary Fig. 6). Interestingly, according to our operando BF-TEM video (Supplementary Movie 1), no bubble formation is observed under potential cycling despite the evolved molecular oxygen signal being clearly detectable in the EEL spectra. This indicates that the diffusion of the evolved O_2_ in the thin liquid is fast enough to avoid gas bubble formation during cycling, since gas bubble formation during gas evolving reactions is a dynamic process of the continuous local release of the supersaturation of dissolved gas near the catalyst surfaces when diffusion or convection are not fast enough^31^.

### Switchable wetting behaviour of cobalt-based oxides

**Fig. 3:**
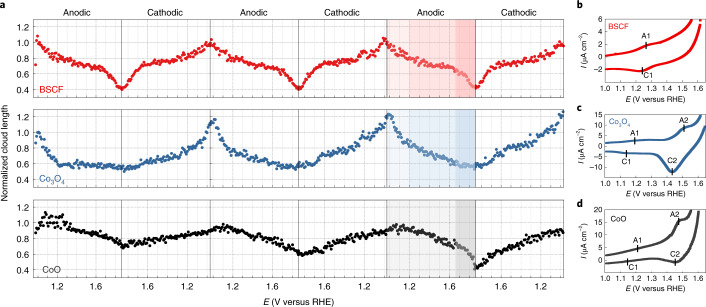
Potential-induced switchable wetting behaviour of cobalt-based oxides. **a**, Plot of normalized cloud length as a function of applied potential for the first three cycles. The cloud length was normalized to its value at 1.0 V versus RHE (initial state). **b**–**d**, Representative cyclic voltammograms of BSCF (**b**), Co_3_O_4_ (**c**) and CoO (**d**) in the potential range 1.0–1.65 V versus RHE. Scan rate, 10 mV s^−1^. The measurements were performed in a microvolume liquid cell. A1/C1 and A2/C2 redox waves are attributed to Co^2+^/Co^3+^ and Co^3+^/Co^4+^, respectively. Note that the polarization curves of cobalt-based oxides in **b**–**d** were acquired from cells that were heavily dropcast with oxide particles to discern the redox features. The full range of polarization curves are shown in Supplementary Fig. 9. Source data

To better understand the origins of the reversible wettability of BSCF, electrochemical liquid-phase TEM was performed on two other oxygen-evolving cobalt-based oxides: CoO and Co_3_O_4_. CoO crystallizes in the rock-salt structure with Co^2+^ ions occupying octahedral sites (O_h_) while Co_3_O_4_ belongs to the spinel crystal structure with cobalt ions having mixed valence of +2 in the tetrahedral sites (T_d_) and +3 in the octahedral sites with a ratio of 1:2. The sequence of the BF-TEM images of BSCF, Co_3_O_4_ and CoO under potential cycling are shown in Supplementary Movies 1–3, respectively, and their CV polarization curves are shown in Supplementary Fig. 7. In all three cases, a similar cloud contrast was formed around the particles at the beginning of the scan and dissolved progressively with the application of the anodic potential. To quantitatively analyse the results and compare the behaviour of the different oxides, the one-dimensional cloud length of each oxide was normalized to the value of the cloud length at the launch of the synchronized imaging with the scan, at 1.0 V versus RHE. Figure 3a depicts the normalized cloud length as a function of applied potential of the first three cycles and reflects the changes in the wetting character during potential cycling. The plots of the first five cycles of the three cobalt-based oxides are shown in Supplementary Fig. 8. Overall, the three oxides show periodic variations in the normalized cloud length with respect to the applied potential, indicating reversible wettability during cycling. The trend is similar in all cases. They show a decreasing cloud length at anodic potentials, up until the highest applied potential, before switching to increasing cloud lengths at cathodic scans. The normalized cloud length curves of BSCF and Co_3_O_4_ exhibit three characteristic regions at each half-cycle at low potentials (<1.2 V versus RHE), intermediate potentials (1.2–1.65 V versus RHE) and high potentials (>1.65 V versus RHE). When the anodic potential is applied, the cloud starts to diminish, indicating a reduction in the hydrophobicity of the surfaces. At the beginning of the intermediate potential region, at ∼1.2 V versus RHE, a distinctive slope flattening occurs. The variation with respect to the applied potential is accompanied by the stabilization of the cloud surrounding the particles, indicating that the surface wetting character of BSCF and Co_3_O_4_ at the intermediate potential range (1.2–1.65 V versus RHE) does not alter substantially and the OH^−^ charge accumulation stabilizes. The slope change at 1.2 V versus RHE results from the surface cobalt redox reaction. In fact, the redox feature (A1/C1) is found in the polarization CV curves of both BSCF and Co_3_O_4_ (Fig. 3b,c, respectively). Moreover, the CV of Co_3_O_4_ shows an additional cobalt redox feature A2/C2 at 1.5 V versus RHE. No considerable change occurs at 1.5 V versus RHE in the Co_3_O_4_ normalized cloud length plot, meaning that the first surface cobalt redox reaction dominates its wetting behaviour. In fact, the two redox features A1/C1 and A2/C2 are attributed to Co^2+^/Co^3+^ and Co^3+^/Co^4+^, respectively^32^. This indicates that Co^2+^/Co^3+^ plays a major role in the wettability characteristics of the cobalt-based oxide catalysts. Although Co^3+^/Co^4+^ redox peaks are more pronounced in the CVs of CoO and Co_3_O_4_, this does not lead to further modification of the cobalt local oxygen-coordination shell^33^. Thus, the reaction does not have an effect on the surface wettability. The abrupt reduction in the normalized cloud length plot of BSCF at the third region, above 1.65 V versus RHE, is linked to the evolution of O_2_. For Co_3_O_4_, no pronounced reduction in the cloud surrounding the particles is seen in the third region. This could be attributed to the fact that Co_3_O_4_ is less OER-active than BSCF^34^. In contrast to the other two oxides, CoO varies almost linearly with respect to the applied potential, with the slope remaining constant during the first half-scans. In other words, the reduction of the hydrophobicity of CoO remains constant across the first redox feature and the accumulation of OH^−^ is continuous across the full potential range. This nearly constant slope could be attributed to the indistinct A1/C1 redox feature, as shown in Fig. 3d, which can be related to the Co^2+^ octahedral coordination in the rock-salt crystal structure. It has been reported that the octahedrally coordinated Co site is inactive and it is not involved in the oxyhydroxide phase transformation^35^. Thus, Co^2+^ ions in octahedral sites may lead to a less pronounced Co^2+^/Co^3+^ redox feature^36^. Finally, both BSCF and Co_3_O_4_ exhibit relatively stable wetting behaviour across the three cycles, with BSCF being particularly stable across the anodic and cathodic scans. For CoO, however, its wettability changes are not as stable as for the other two oxides as shown in Supplementary Fig. 8c. The average slopes are getting steeper as the cycle number increases. This could be related to the previously reported irreversible CoO to Co_3_O_4_ transformation at the initial cycles^32^ that causes the interfacial capacitance to change.

### Proposed mechanism of wetting behaviour

**Fig. 4:**
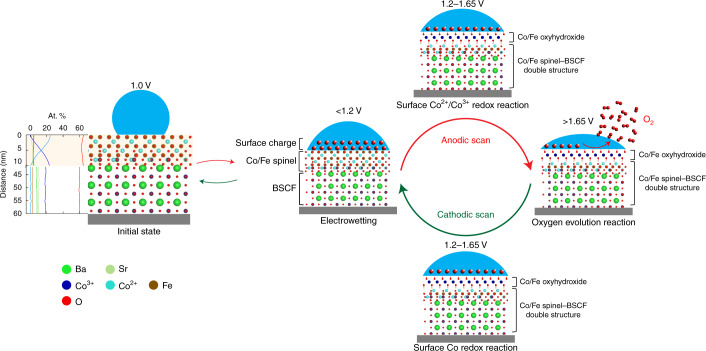
Proposed mechanism of switchable wetting at the BSCF oxide surface under potential cycling. Electrowetting induces a hydrophobic to hydrophilic transition on the initial Co/Fe spinel surface on BSCF. The EELS quantification profile of all elements in BSCF is shown next to the schematic of the initial state. Zero distance corresponds to the surface. Cobalt ions with +2 oxidation state are present at the BSCF surface while the valence of cobalt in bulk is +3 (as expected in the perovskite). The bulk atomic percentages correspond well to the stoichiometry of Ba_0.5_Sr_0.5_Co_0.8_Fe_0.2_O_3−*δ*_. Co/Fe oxyhydroxide forms at the Co/Fe spinel surface at 1.2 V during anodic scan, altering the effect of electrowetting. The surface oxyhydroxide further catalyses the OER at higher potential so the surrounding liquid becomes thinner. The process is reversible.

A mechanism of switchable wetting detailed for the highly active BSCF under potential cycling is schematically illustrated in Fig. 4. The suggested potential-dependent wetting transition of BSCF at the low potential region <1.2 V versus RHE can be explained by the theory of electrowetting as defined by the Young–Lippmann equation^13^:

1 $$({\cos\theta - \cos\theta _{\mathrm{eq}}}) \propto {\int}C(E)E{\mathrm{d}}E$$

This equation relates the difference of the contact angle *θ* after application of the electric potential *E* to the contact angle at equilibrium (at 1.0 V versus RHE in our case), *θ*_eq_, with the potential-dependent capacitance at the solid–liquid interface, *C(E)*. It indicates that on application of the electric potential a thin layer of OH^−^ ions accumulates at the interface, giving rise to interface capacitance that results in a contact angle modification. In practice, this equation implies an overall reduction of the contact angle under the applied potential, providing a qualitative explanation of the reduction of the hydrophobicity and a transition towards a hydrophilic character in BSCF at the low potential range. At 1.2 V versus RHE, the change of slope is related to the surface Co^2+^/Co^3+^ redox reaction in tetrahedral coordination^35,37^. This redox feature has been attributed to reversible oxyhydroxide (CoOOH) phase transformation, which for the Co^2+^ in spinel proceeds with the electrochemical reaction Co_3_O_4_ + OH^−^ + H_2_O ↔ 3CoOOH + *e*^−^ at 1.22 V versus RHE^33,36^. The detailed EELS quantification profile of all elements in BSCF (Fig. 4) confirms the predominance of Co^2+^ ions at the surface with a characteristic increase in the Co^2+^ concentration from 0 at.% at the inner bulk to ∼20 at.% at the surface (light blue profile). The full range of EEL spectra are shown in Supplementary Fig. 10. We have also previously shown that the perovskite BSCF particles possess a Co/Fe spinel surface with reduced cobalt valence which enables the Co(Fe)OOH phase transformation^38^. The spinel–perovskite double structure and its stability during cycling are confirmed by operando selected-area electron diffraction measurements (Supplementary Fig. 11 and Supplementary Movie 4), while the subtle changes of the {113} spinel reflections indicate the restructuring of the spinel surface during cycling. The formation of Co(Fe)OOH at the surface modifies the adsorption of OH^−^ ions at the interface, leading to alteration of the interface capacitance. Additionally, taking into account the previously reported intrinsic oxyhydroxide phase hydrophilicity in nickel-based catalysts^39^, we conclude that BSCF transitions towards a hydrophilic character at 1.2 V versus RHE, which remains relatively stable until 1.65 V versus RHE. Similar electrochemically induced modification of intrinsic surface activity has also been demonstrated on oxidation of copper surface^10^ and liquid gallium^40^ by application of electric potential.

At the third region, above 1.65 V versus RHE, the evolution of O_2_ results in a globally thinner liquid layer. The influence of the OER on local liquid movement can be attributed to two factors. First, the increase in the volume of the gas-filled region compresses the liquid layer during the OER. As the cell is not completely filled with liquid, gas is also present in the cell. The evolved molecular O_2_ from the catalytic oxide surface dissolves and diffuses through the thin liquid layer, and once the molecular oxygen reaches the gas–liquid interface, it precipitates and induces the growth of the volume in the gas-filled region. The growth of the gas region thus attenuates the thickness of the liquid. Second, the liquid electrolyte surrounding the surface of the particles is consumed during the OER, which leads to a reduced liquid layer. Both effects act synergistically and contribute to liquid movement at potentials where the OER takes place.

Further, our results help access how the liquid moves under the OER. The relative thickness that is extracted from the low-loss EEL spectra is indicative of the thickness of the cell in its entirety along the *z* direction (parallel to the electron beam) while the normalized cloud length provides information about the in-plane liquid movement (perpendicular to the electron beam). In the OER regime the normalized cloud length is continuously decreasing on the anodic scan, whereas the relative thickness reaches a plateau and remains unaltered (as shown in Supplementary Fig. 12). This means that the liquid thickness is reduced in the in-plane direction (due to the consumption of liquid surrounding the particles) while the overall thickness of the cell does not change.

## Conclusions

We have demonstrated switchable wetting behaviour at oxygen-evolving catalyst surfaces, specifically cobalt-based oxides, on the basis of relating the contrast seen in electrochemical liquid-phase TEM to the movement of the liquid surrounding the particles. The alteration of the surface wetting character shows three distinct regions during cycling, which are related to electrowetting and surface reconstruction mechanisms. At low applied potential (<1.2 V versus RHE), electrowetting and potential-dependent interfacial capacitance induce a hydrophobic to hydrophilic transition. The formation of an oxyhydroxide phase at the surfaces at 1.2 V versus RHE also leads to the attainment of a hydrophilic wetting character. For potentials larger than 1.65 V versus RHE, the surface oxyhydroxide further catalyses the adsorbed hydroxide ions at the solid–liquid interface to form molecular oxygen, as verified by operando EELS measurements. Liquid-phase TEM can provide unique insights of wetting dynamics in real time, opening a pathway to better understand the solid–liquid interface interactions. Moreover, we demonstrated its capability to detect the products of catalytic reactions at a single-particle level.

## Methods

### Materials

BSCF particles were synthesized using a nitrate combustion method reported previously^41,42^. X-ray diffraction of the synthesized BSCF revealed a perovskite structure (space group, *Pm*−3*m*) with a lattice parameter of 3.99 Å (refs. ^41,42^). CoO (cobalt(ii) oxide, 99.99% trace metals basis, Sigma-Aldrich) and Co_3_O_4_ (cobalt(ii,iii) oxide, nanopowder <50 nm particle size, 99.5% trace metals basis, Sigma-Aldrich) were used.

### Working electrode preparation

Customized microelectromechanical (MEMS) fabricated chips patterned with three ultrathin platinum electrodes were used to deposit the catalysts and perform the operando TEM experiments. The catalyst suspensions were prepared by dispersing cobalt-based oxide powders in isopropanol. The suspensions were then dropcast on to the electrodes on the MEMS chips and left to dry (minutes).

### Electrochemical measurements

A potentiostat (Bio-Logic SP-300) with an ultralow-current cable was used to perform electrochemical operations. All the electrochemical measurements were performed in a three-electrode configuration. The geometric surface area of the platinum working electrode is 0.001 cm^2^. For the operando experiments performed on the MEMS platinum chips, one of three ultrathin platinum electrodes is used as a quasi-reference electrode. This platinum quasi-reference electrode was calibrated as 0.2 ± 0.04 V versus SHE by measuring the potential against an external alkaline reference electrode (0.1 M KOH filled, RE-61AP, BASi) in 0.1 M KOH solution. The ultrathin platinum electrode with a larger surface area on the MEMS chips was used as a counterelectrode.

### Liquid-phase TEM set-up

Liquid-phase TEM analysis was performed using a liquid-electrochemistry holder (Hummingbird Scientific). First, the customized platinum chips (top chips) and 1 µm spacer chips (bottom chips) were air-plasma treated for 2 min to functionalize the surfaces. After the oxide suspensions were dropcast on the top chips, both top and bottom chips were assembled on to the TEM holder. Finally, 0.1 M KOH was injected into the liquid cell through the flow tubes.

### Operando TEM and selected-area electron diffraction analyses

The operando TEM and selected-area electron diffraction (SAED) measurements were performed in a JEOL 2200FS TEM operated at 200 kV. Zero-loss energy-filtering was performed with the in-column omega energy filter to remove inelastic scatting of the system that includes the SiN_*x*_ membranes and liquid. A 12 eV energy-selecting slit was centred on the zero-loss peak in the EELS for the operation of energy-filtered TEM imaging and SAED acquisition. For the TEM imaging, a direct electron detector (DE-16, Direct Electron) with high sensitivity and temporal resolution was used to record TEM images using StreamPix software package, while the CVs were simultaneously recorded. The frame rate of the recording was set to 20 f.p.s., and the camera binning was set 2 to give a final image size of 2,048 × 2,048 pixels. The raw TEM images were averaged for every 10 frames, providing TEM image sequences with 2 f.p.s. SAED patterns during cycling were acquired on a CMOS detector (OneView, Gatan) with a frame exposure time of 0.5 s.

### Image segmentation

The TEM images were segmented to calculate the one-dimensional cloud length. Each frame of the TEM images was divided into three classes for the particle, cloud and background using the plugin Trainable Weka Segmentation in FIJI image-processing software. The classifier was trained with a random forest algorithm. After the classifier was trained, it was applied to the TEM image sequences to classify the three regions in the images. Examples of the segmented images of three cobalt oxides are shown in Supplementary Fig. 13. Once the images were segmented, the targeted region, for example, the cloud, was thresholded to calculate the area. A rectangle (red in Supplementary Fig. 13) perpendicular to the particle surface was drawn across the particle and cloud. The length of the cloud within this rectangle was calculated.

### EELS characterization

EELS data were acquired in STEM mode. The STEM-EELS data acquisition was performed using a spherical aberration (Cs)-corrected TEM (Titan Themis 60-300, ThermoFisher Scientific) equipped with a high brightness Schottky field emission source (XFEG). All experiments were performed at 300 kV. Energy-loss spectra were acquired with a post column electron energy-loss spectrometer (GIF Quantum ERS, Gatan) at convergence and collection angles of 28 and 19.8 mrad, respectively. The energy dispersion was 0.1 eV per channel, giving an energy resolution of ∼1.1 eV. Operando EEL spectra were acquired in spot mode using time-series and dual-EELS modes. The low loss energy offset was set to 0 eV to enable post-acquisition zero-loss peak and plural scattering correction of the core-loss spectral datasets. The background of the core-loss spectra was subtracted using Gatan Microscopy Suite software.

### EELS quantification

A JEOL F200 CFEG (S)TEM fitted with a K3 GIF Continuum and operated at 200 kV was used to acquire EEL spectra from BSCF particles dropcast on to lacey carbon support film for elemental quantification. The probe current was 200 pA during acquisition, and the convergence angle and collection angle were 8 and 16 mrad, respectively. Spectrum image (SI) (Supplementary Fig. 10a) data were acquired in dual-EELS mode using a K3 electron-counting detector at spectrometer energy dispersions of 0.45 eV per channel. Low-loss and high-loss spectral acquisition times were 1 and 5 ms, respectively, giving a total dwell time of 6 ms per SI pixel. Zero-loss peak lock was enabled. Two-dimensional array spectrum images were acquired in multiple passes, giving a total accumulated acquisition time of 30 ms per SI pixel. Line profile spectrum images were calculated from the two-dimensional array datasets, post-acquisition, to improve the signal-to-noise ratio for EELS quantification. Standards-based quantification with plural scattering correction was used to determine chemical concentration profiles for oxygen, iron, cobalt, barium and strontium. Concurrent standards were used for cobalt quantification, allowing separate concentration profiles to be determined for Co(ii) and Co(iii). Reference spectra for Co(ii) and Co(iii) were acquired from CoO and LiCoO_2_ powders.

## Supplementary information

Supplementary Information Supplementary Figs. 1–13 and captions for Movies 1–4.

Supplementary Video 1 BF-TEM images of BSCF under potential cycling.

Supplementary Video 2 BF-TEM images of BSCF of Co_3_O_4_ under potential cycling.

Supplementary Video 3 BF-TEM images of BSCF of CoO under potential cycling.

Supplementary Video 4 SAED patterns of BSCF under potential cycling.

## Data Availability

The data supporting the findings of this study are available in the paper and its Supplementary Information. Extra data are available from the corresponding authors on reasonable request. Source data are provided with this paper.

## Source data

Source Data Fig. 1 Statistical Source Data.
Source Data Fig. 2 Statistical Source Data.
Source Data Fig. 3 Statistical Source Data.
