# Supplementary material for: Switchable wetting of oxygen-evolving oxide catalysts
Source: Nat Catal. 2021 Dec 30;5(1):30–6. doi: 10.1038/s41929-021-00723-w (PMC8799463; doi:10.1038/s41929-021-00723-w)
Supplement: Supplementary file 1 — Supplementary Figs. 1–13 and captions for Movies 1–4. [file 41929_2021_723_MOESM1_ESM.pdf]

nature portfolio

https://doi.org/10.1038/s41929-021-00723-w

Supplementary information

Switchable wetting of oxygen-evolving oxide catalysts

In the format provided by the
authors and unedited

Supplementary Information

Switchable wetting of oxygen evolving oxide catalysts

Tzu-Hsien Shen,[1] Liam Spillane[2], Jiayu Peng[3], Yang Shao-Horn[3,4,5], Vasiliki Tileli[1]*

[1]Institute of Materials, École Polytechnique Fédérale de Lausanne, CH–1015 Lausanne, Switzerland

[2]Gatan Inc., Pleasanton, California 94588, United States

[3]Department of Materials Science and Engineering, Massachusetts Institute of Technology, Cambridge, Massachusetts 02139, United States

[4]Department of Mechanical Engineering, Massachusetts Institute of Technology, Cambridge, Massachusetts 02139, United States

[5]Research Laboratory of Electronics, Massachusetts Institute of Technology, Cambridge, Massachusetts 02139, United States

This supporting information includes

Supplementary Figures 1-13

Captions for Supplementary Movies 1-4

Supplementary Figures

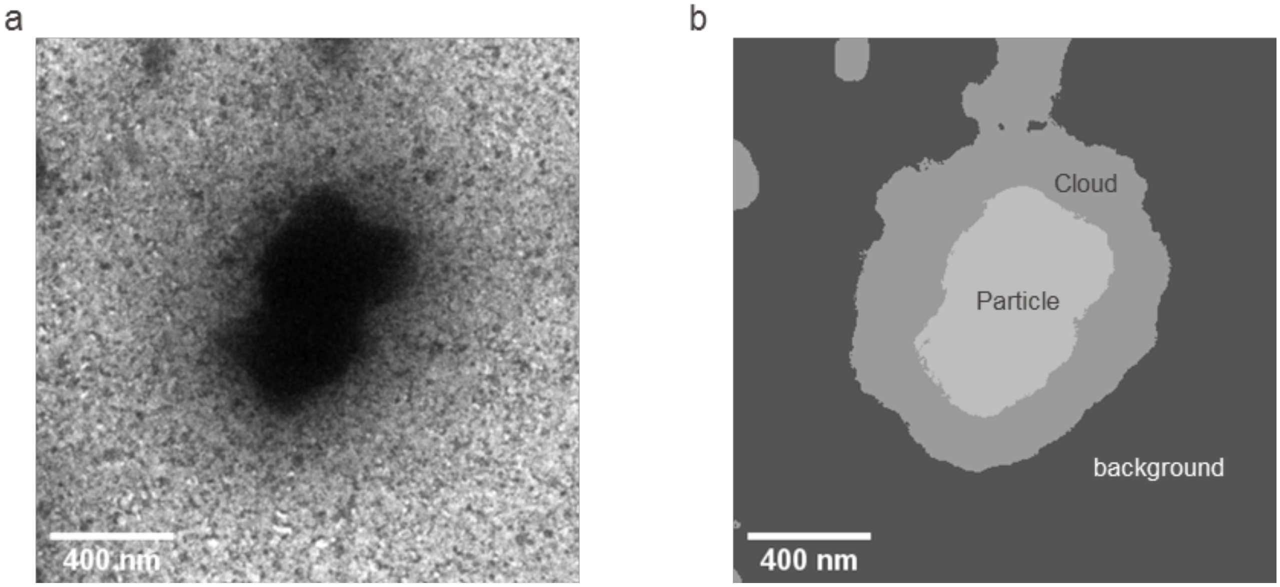

Supplementary Fig. 1 | Segmented BF-TEM image of a BSCF particle surrounded by 0.1 M KOH solution. a, first frame of BF-TEM image. b, segmented image of particle, cloud, and background.

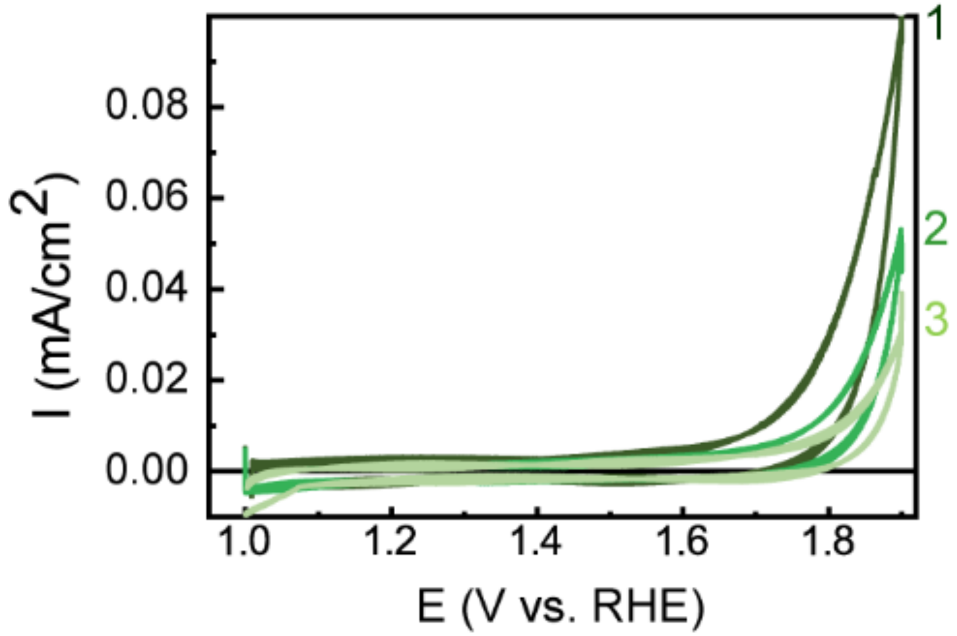

Supplementary Fig. 2 | Polarization curves of the three CV cycles of BSCF during the operando EELS experiments. The scan rate is 20 mV/s. The reduction of the current density from the first to third cycles is due to the drift of quasi-reference Pt electrode.

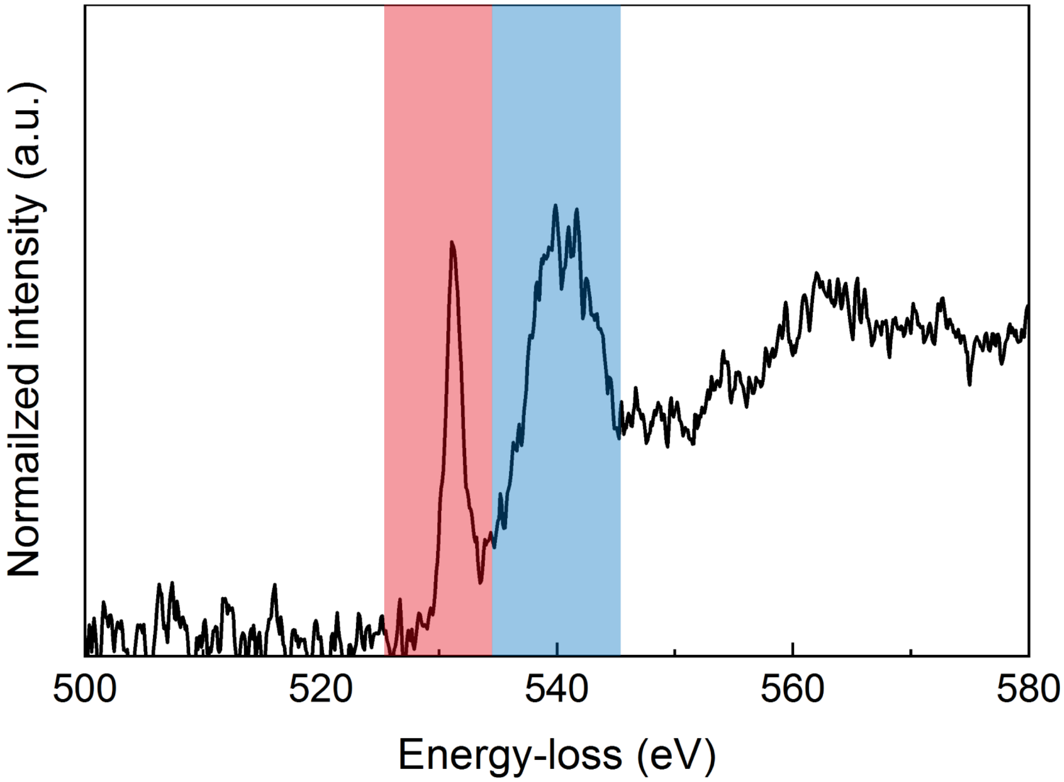

Supplementary Fig. 3 | Core-loss O K EELS. The O_2 peak intensity ratio is defined as the molecular peak intensity in the range 529-534 eV (red shaded region) divided by the background intensity ranging from 535 to 545 eV (blue shaded region).

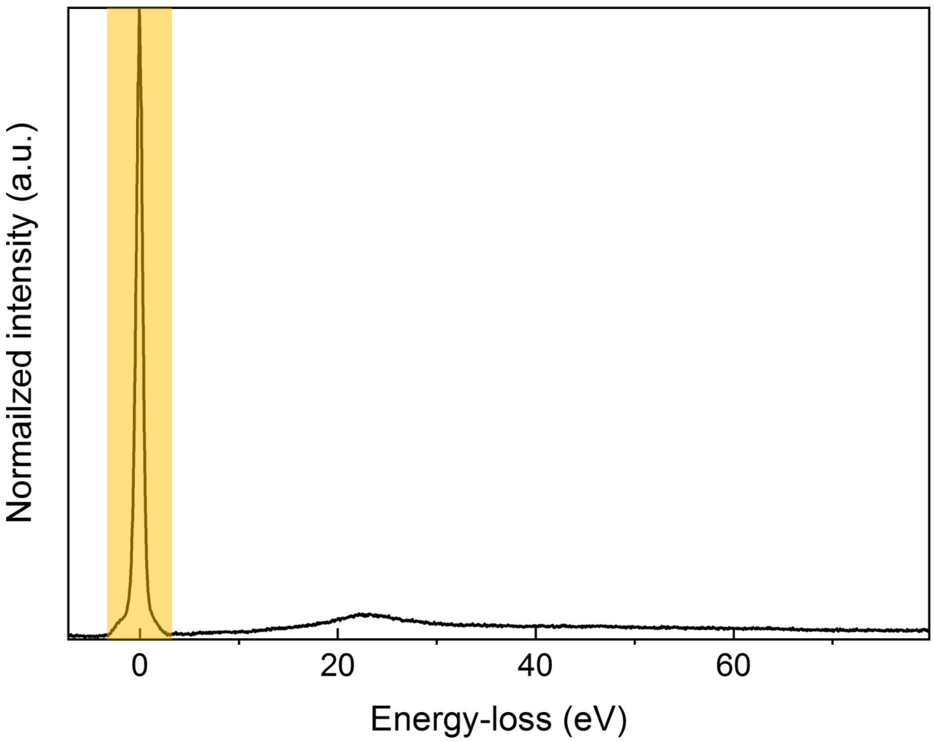

Supplementary Fig. 4 | Low-loss EELS. The relative thickness (t/λ) is defined as $-\ln\left(\frac{\text{intensity of the area of the zero−loss peak (yellow shaded region)}}{\text{entire EEL spectrum intensity}}\right)$. This measurement gives an indication of the total thickness of the liquid cell.

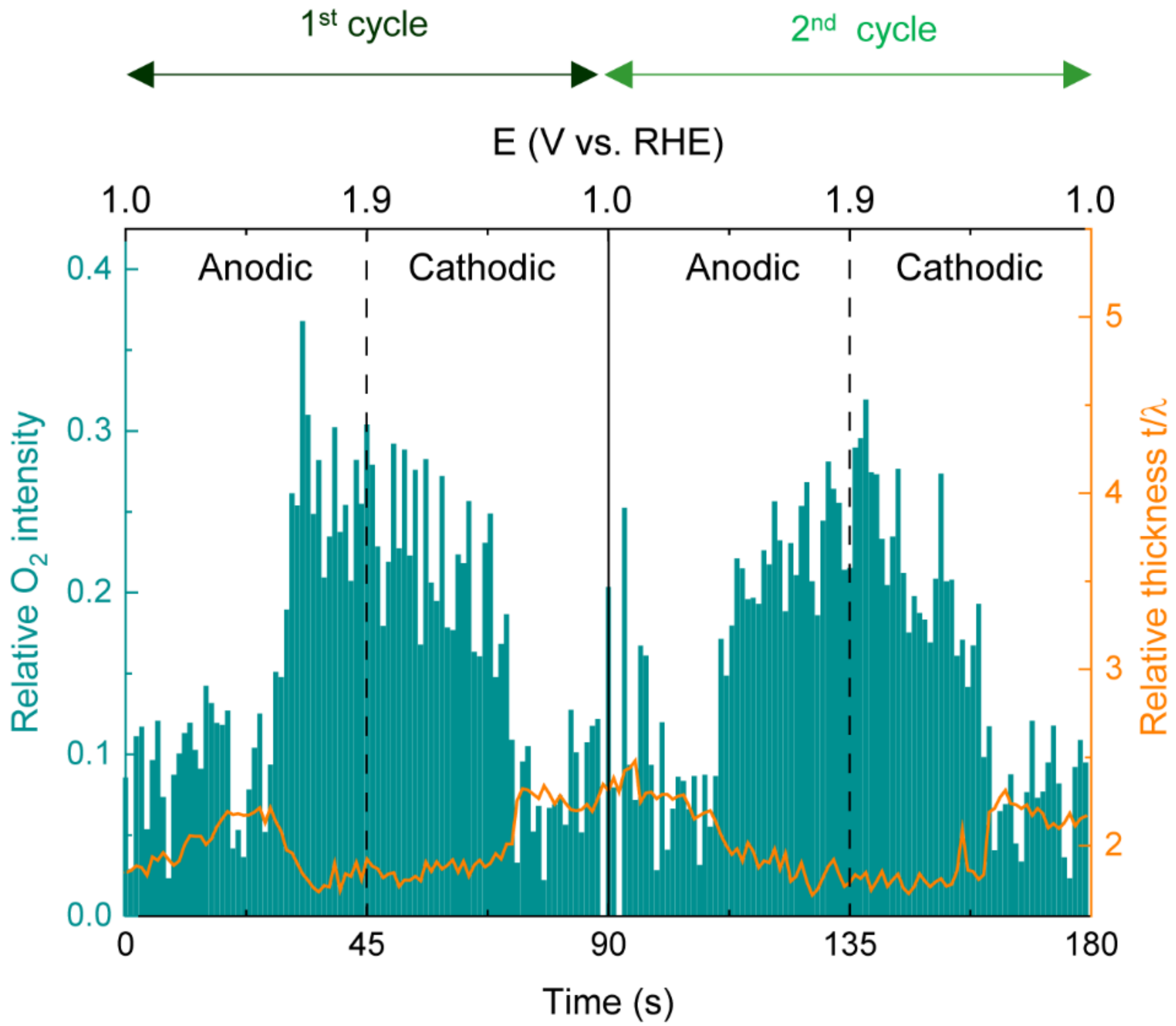

Supplementary Fig. 5 | Operando EELS analysis of Co_3O_4 under potential cycling. Plot of relative O_2 intensity (green) and thickness (orange curve) as a function of elapsed time (bottom) and applied potential (top) corresponding to two CV cycles.

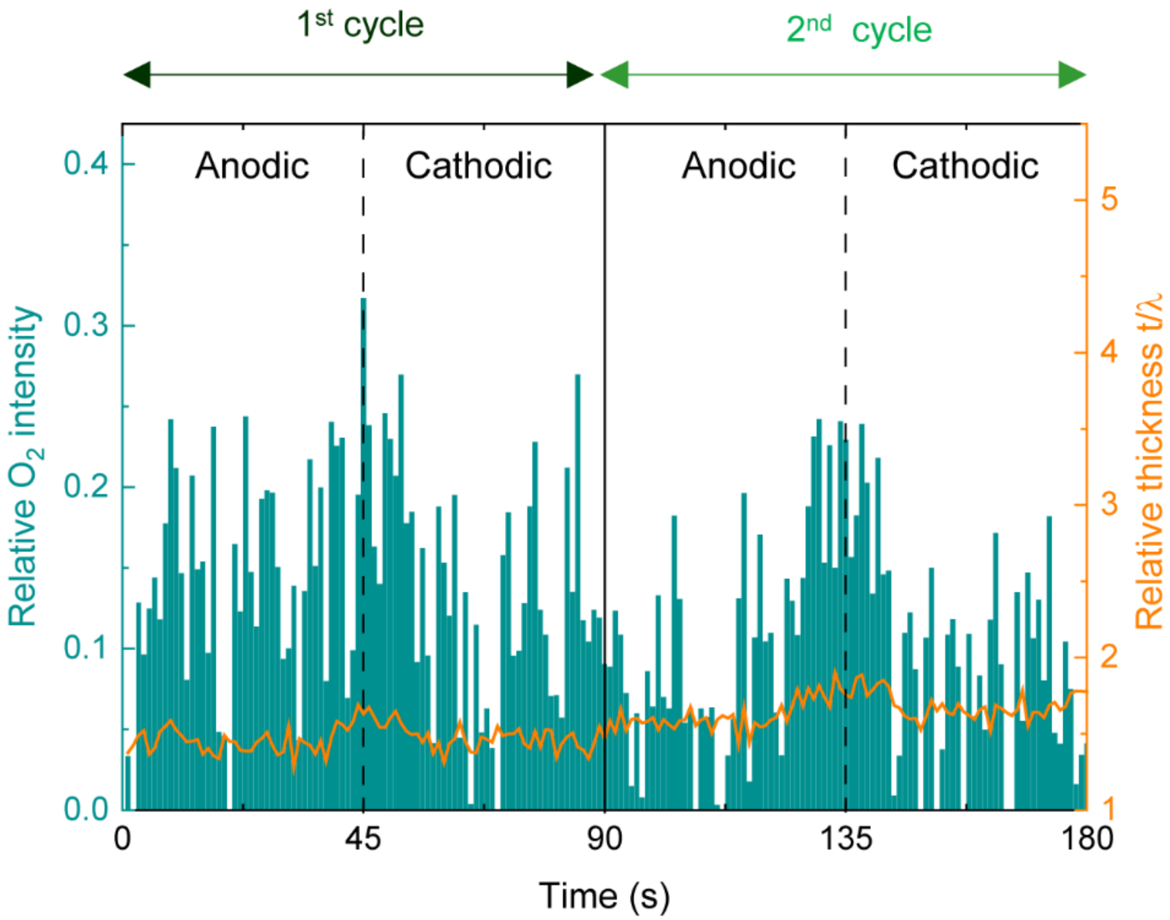

Supplementary Fig. 6 | Operando EELS analysis of the Pt electrode under potential cycling. Plot of O_2 peak intensity (green) and relative thickness (orange curve) as a function of elapsed time (bottom) and applied potential (top) corresponding to two CV cycles.

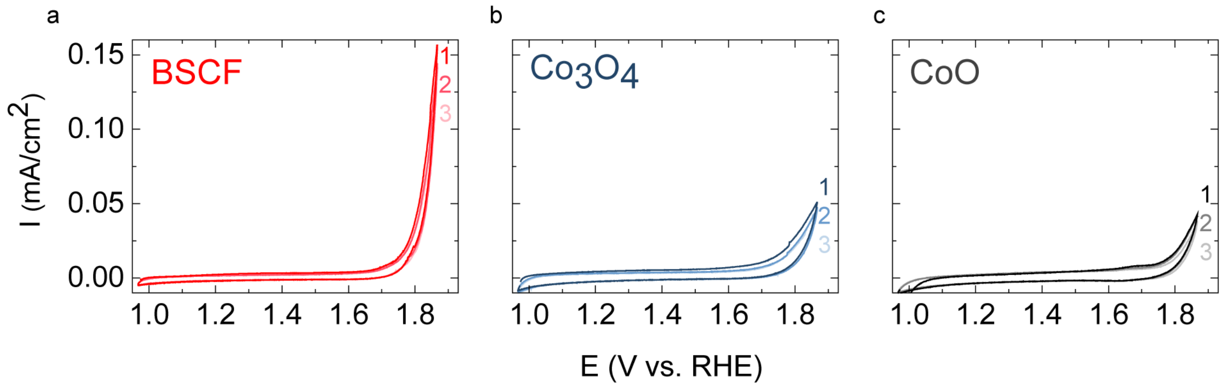

Supplementary Fig. 7 | The polarization curves of CV of BSCF (red), Co_3O_4 (blue), and CoO (black) in liquid-cell enclosure. The scan rate is 20 mV/s.

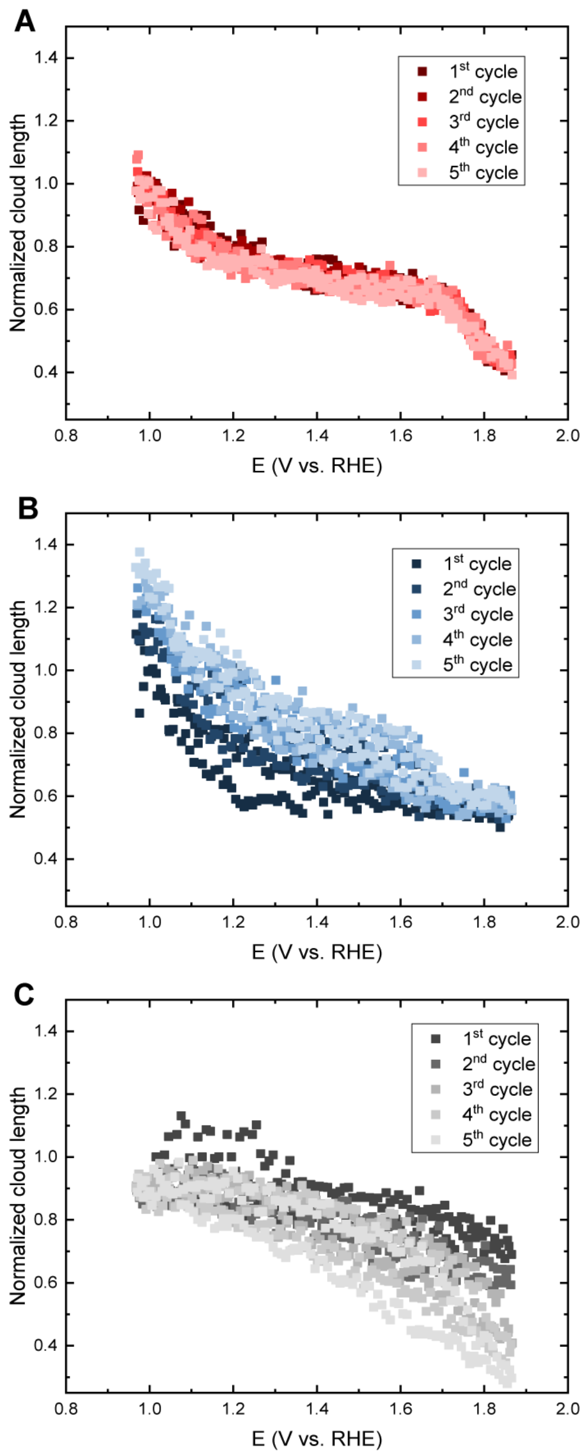

Supplementary Fig. 8 | Plot of normalized cloud length as a function of applied potential for the complete 5 cycles. a, BSCF. b, Co_3O_4. c, CoO.

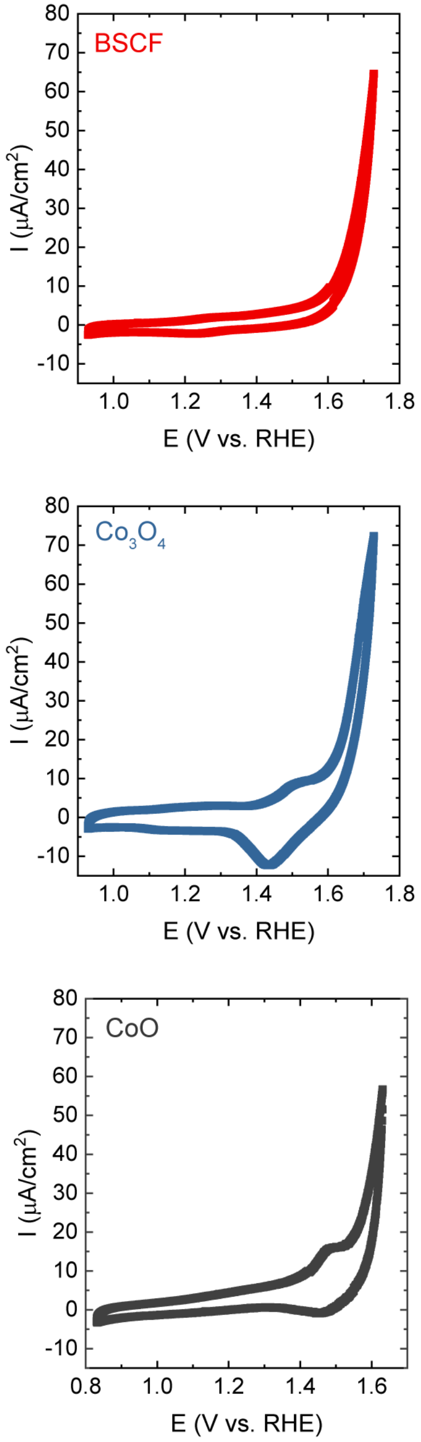

Supplementary Fig. 9 | The full range of polarization curves in Fig. 3b-d. BSCF (red), Co_3O_4 (blue), and CoO (black) in liquid-cell enclosure. The scan rate is 10 mV/s. It is noted that the OER currents of different oxides in the CVs acquired in the liquid cell TEM measurements are not comparable due to variations with the particle loading on the Pt electrode and the volume of the liquid solution.

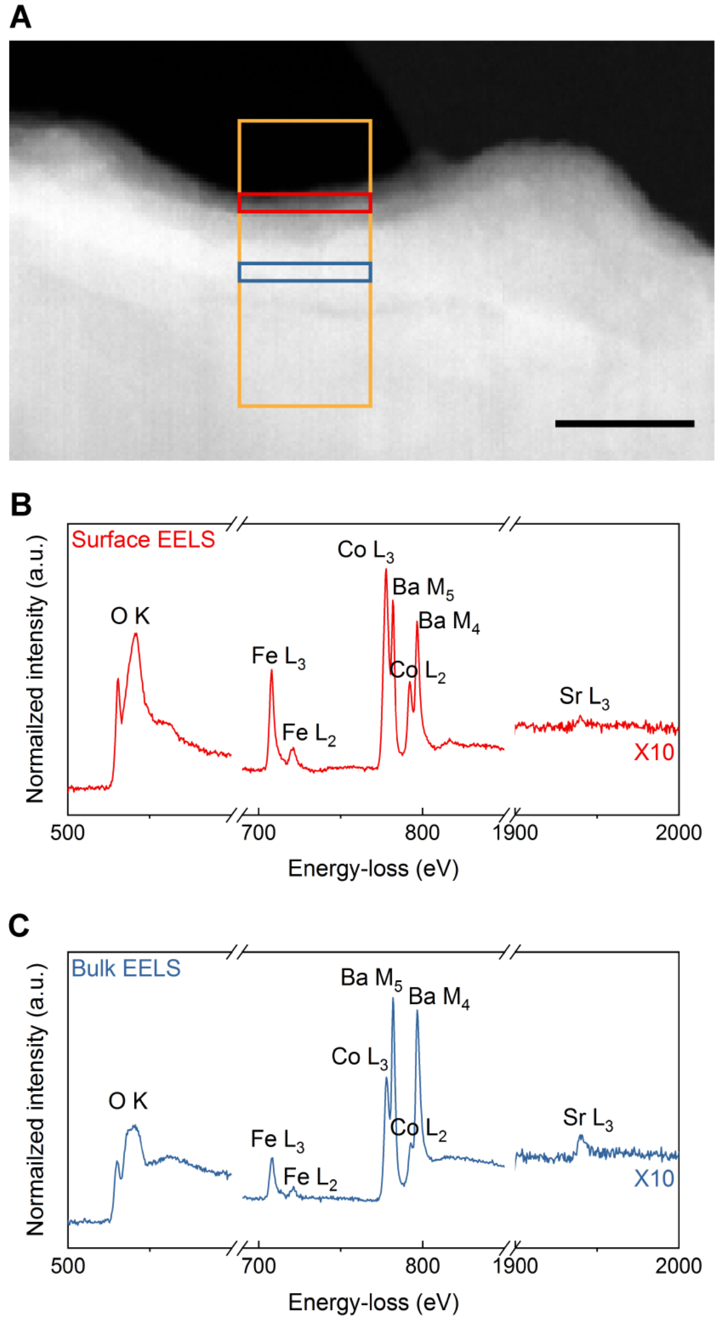

Supplementary Fig. 10 | Full energy-loss range EELS spectra involving Ba, Sr, Co, Fe, and O for EELS elemental quantification. (a) HAADF-STEM image of BSCF surface. The orange rectangle indicates the region in which the 2D array spectrum image was acquired. The scale bar is 100 nm. (b) EELS from the surface of BSCF (red rectangular region in a). (c) EELS from the bulk of BSCF (blue rectangle in c). Background is removed and plural scattering is deconvolved in both EEL spectra. The normalized intensity in spectrum region range from 1900-2000 eV was increased by an order of magnitude.

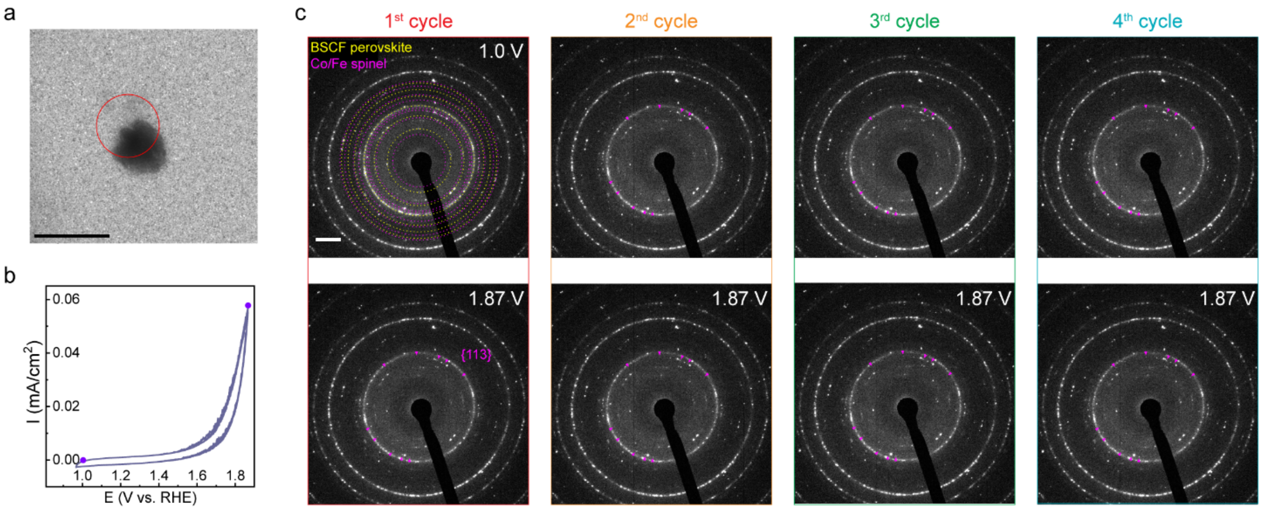

Supplementary Fig. 11 | Operando selected area diffraction analysis of BSCF under potential cycling. (a) BF-TEM image of BSCF particle on Pt electrode. The red ring indicates the position and size of the selected area aperture. The scale bar is 1 µm. (b) Polarization curves of the 4 cycles of the experiment. The two purple points indicate the potentials at which SAED patterns in c are extracted for (c). The scan rate is 20 mV/s. (c) Frames of SAED patterns at specific potentials. Yellow and magenta rings indicate BSCF perovskite and Co/Fe spinel reflections, respectively. The magenta arrows indicate the {113} spinel reflections. The bright rings, which do not belong to perovskite or spinel, are associated with the underlying Pt polycrystalline thin film electrode. The scale bar is 2 nm^{-1}.

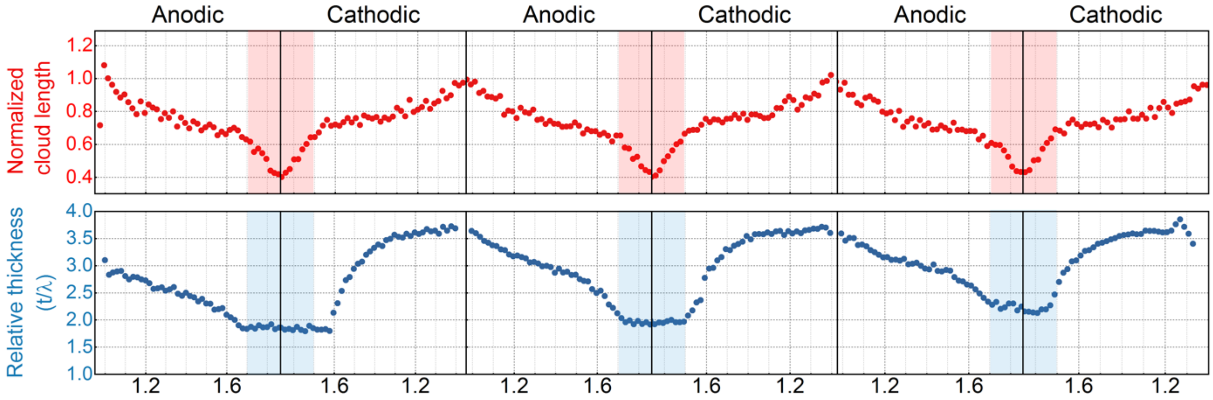

Supplementary Fig. 12 | Comparison of normalized cloud length and relative thickness under potential cycling. The shaded regions indicate oxygen evolving conditions.

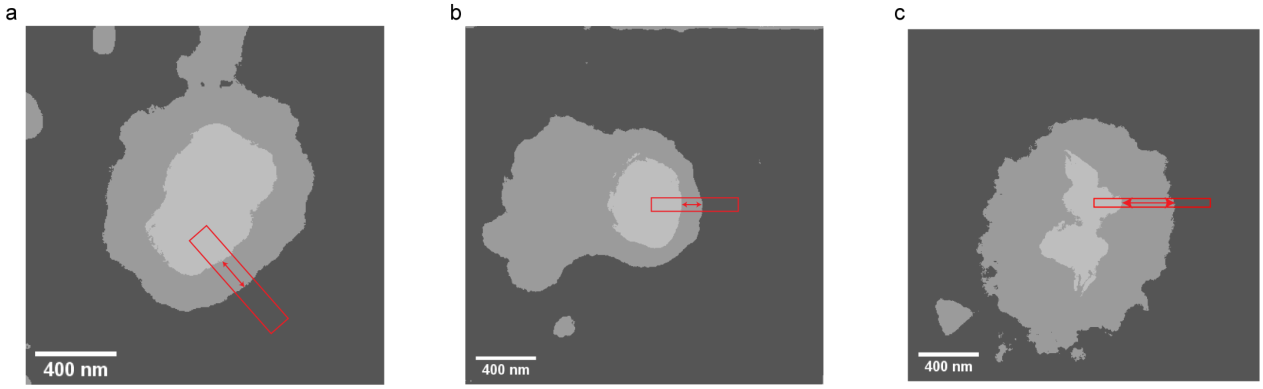

Supplementary Fig. 13 | Segmentation of TEM images of three Co-based oxides. **a**, BSCF. **b**, Co_3O_4. **c**, CoO. The TEM image sequences are segmented into three categories: Background (dark grey), Cloud (medium grey), and Particle (light grey). The red rectangles are used for the determination of the parameter cloud length (indicating with red arrows). The areas of cloud and particle within the rectangles were calculated and then the areas were divided by the width of the rectangles. The cloud lengths can be determined.

Captions of Supplementary Movies

The Supplementary Movies of BF-TEM and SAED operando acquisitions were recorded at 2, and 0.5 frames per second (fps) respectively. All movies were sped up to four times for visualization. The BF-TEM image and SAED pattern sequences are shown in synchronization with the cyclic voltammograms.

Supplementary Movie 1 | Operando BF-TEM imaging of BSCF during 1st to 5th cycles

Supplementary Movie 2 | Operando BF-TEM imaging of Co_3O_4 during 1st to 5th cycles

Supplementary Movie 3 | Operando BF-TEM imaging of CoO during 1st to 5th cycles

Supplementary Movie 4 | Operando SAED of BSCF during 1st to 4th cycles
